# Supplementary material for: Enhancing Cardiovascular Risk Prediction with a Simplified Carotid IMT Protocol: Evidence from the IMPROVE Study
Source: Biomedicines. 2025 Feb 26;13(3):584. doi: 10.3390/biomedicines13030584 (PMC11940431; doi:10.3390/biomedicines13030584)
Supplement: Supplementary file 1 [file biomedicines-13-00584-s001.zip › biomedicines-3466293-supplementary.pdf]

SUPPLEMENTARY MATERIALS

SUPPLEMENTARY TABLES

Table S1. Ultrasonographic summary measures considered in the present study.

**1CC-IMT$_{mean-of-2-max}$**:	the average of the maximal IMT measures detected in the **1st cm** of left and right **common carotids**, considering the **lateral projection** only (two points in total) Figure 1, Panel A.
**BIF-IMT$_{mean-of-2-max}$**:	the average of the maximal IMT measures detected in left and right **bifurcations**, considering the **lateral projection** only (two points in total) Figure 1, Panel B.
**IMT$_{mean-max}$**:	the average of the maximal IMT measures detected in four segments (internal, bifurcation, 1st cm of common carotid and the remaining of the common carotid) in both carotid arteries (eight points in total).
**PF-CC-IMT$_{mean}$**:	the average of all the measures taken in **plaque-free areas** (IMT ≤ 1.5 mm) of the left and right common carotids; this is an ultrasound variable usually employed in published studies [1] and downgraded in the last international guidelines [2].

Table S2. Details on the incident events.

**Sudden Death**	8
**AMI**	74
**Coronary bypass**	12
**Angina pectoris**	83
**Angioplasty stent**	32
**Stroke**	81
**TIA**	47
**Claudication**	21
**Peripheral bypass**	9
**Total**	367

Table S3. AUC increase (95% CI) for both combined events and hard events.

	AUC increase combined events (95% CI)	AUC increase Hard events (95% CI)
**1CC-IMT$_{mean-of-2-max}$**	0.017 (0.003; 0.031)	0.020 (-0.003; 0.198)
**BIF-IMT$_{mean-of-2-max}$**	0.009 (-0.007; 0.025)	0.006 (-0.012; 0.995)
**IMT$_{mean-max}$**	0.029 (0.009; 0.049)	0.028 (0.001; 0.116)
**PF-CC-IMT$_{mean}$**	0.007 (-0.008; 0.021)	0.012 (-0.019; 0.891)

Hard events: sudden death plus acute myocardial infarction plus stroke; CI: Confidence Interval; 1CC: 1stcm of the common carotid; BIF: bifurcation. Definitions of the ultrasonographic variables are reported in Table S1.

Table S4. Time-dependent ROC AUC computed by adding each risk factor to a hypothetical score computed by the other five factors.

Risk factors included in SCORE2/SCORE2-OP	ROC AUC for individual risk factor	Total AUC*	AUC increase (95% CI)	P-value
**Age**	0.616	0.661	0.016 (-0.004; 0.056)	0.12
**Sex**	0.632		0.012 (-0.005; 0.043)	0.15
**LDL-C**	0.602		0.004 (-0.002; 0.016)	0.20
**HDL-C**	0.621		0.001 (-0.011; 0.025)	0.80
**Smoke**	0.624		0.012 (-0.006; 0.048)	0.21
**SBP**	0.607		0.002 (-0.004; 0.014)	0.55

*ROC AUC including all six factors. ROC AUCs were estimated at 10 years of follow-up. AUC: Area Under the Curve; CI: Confidence Interval; LDL-C: Low-Density Lipoprotein Cholesterol; HDL-C: High-Density Lipoprotein Cholesterol; SBP: Systolic Blood Pressure.

Table S5. Quintiles boundaries of ultrasonographic variables presented in Figure S2.

	1st quintile	2nd quintile	3rd quintile	4th quintile	5th quintile
$IMT_{mean-max}$	(0.66; 0.99)	(0.99; 1.11)	(1.11; 1.25)	(1.25; 1.45)	(1.45; 2.81)
$1CC-IMT_{mean-of-2-max}$	(0.61; 0.85)	(0.86; 0.93)	(0.93; 1.00)	(1.00; 1.12)	(1.12; 3.10)
$BIF-IMT_{mean-of-2-max}$	(0.57; 0.97)	(0.97; 1.18)	(1.18; 1.40)	(1.40; 1.76)	(1.76; 4.63)
$PF-CC-IMT_{mean}$	(0.50; 0.64)	(0.64; 0.67)	(0.67; 0.70)	(0.70; 0.73)	(0.73; 0.98)

Definitions of the ultrasonographic variables are reported in Table S1.

SUPPLEMENTARY FIGURES

Figure S1. Flow diagram: participants selection.

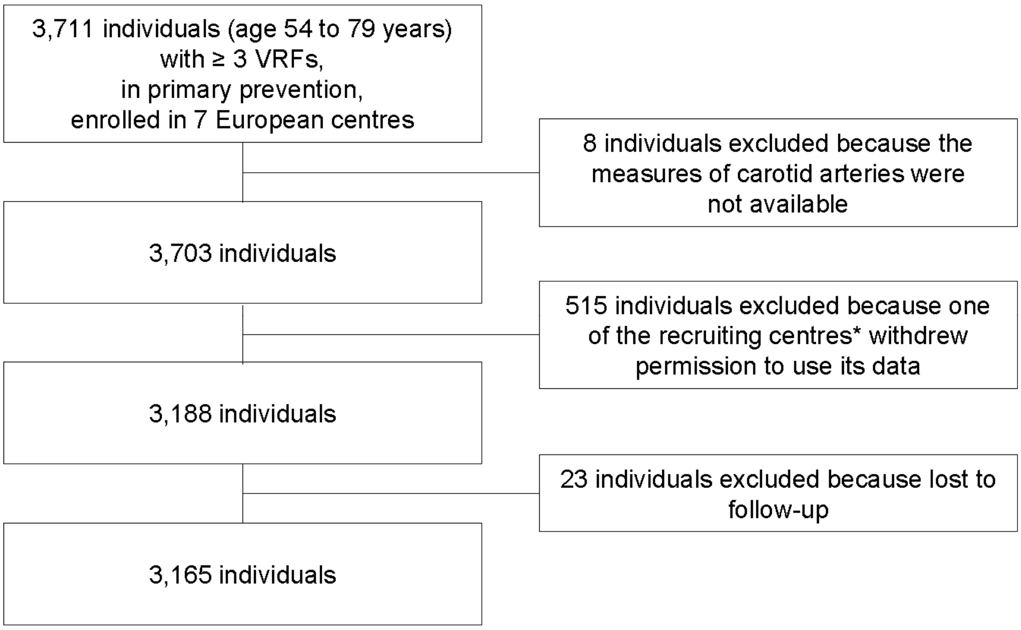

*Institute of Public Health and Clinical Nutrition at the University of Eastern Finland; Kuopio, Finland.

Figure S2. Relation between ultrasonographic variables (quintiles) and HRs for the combined event.

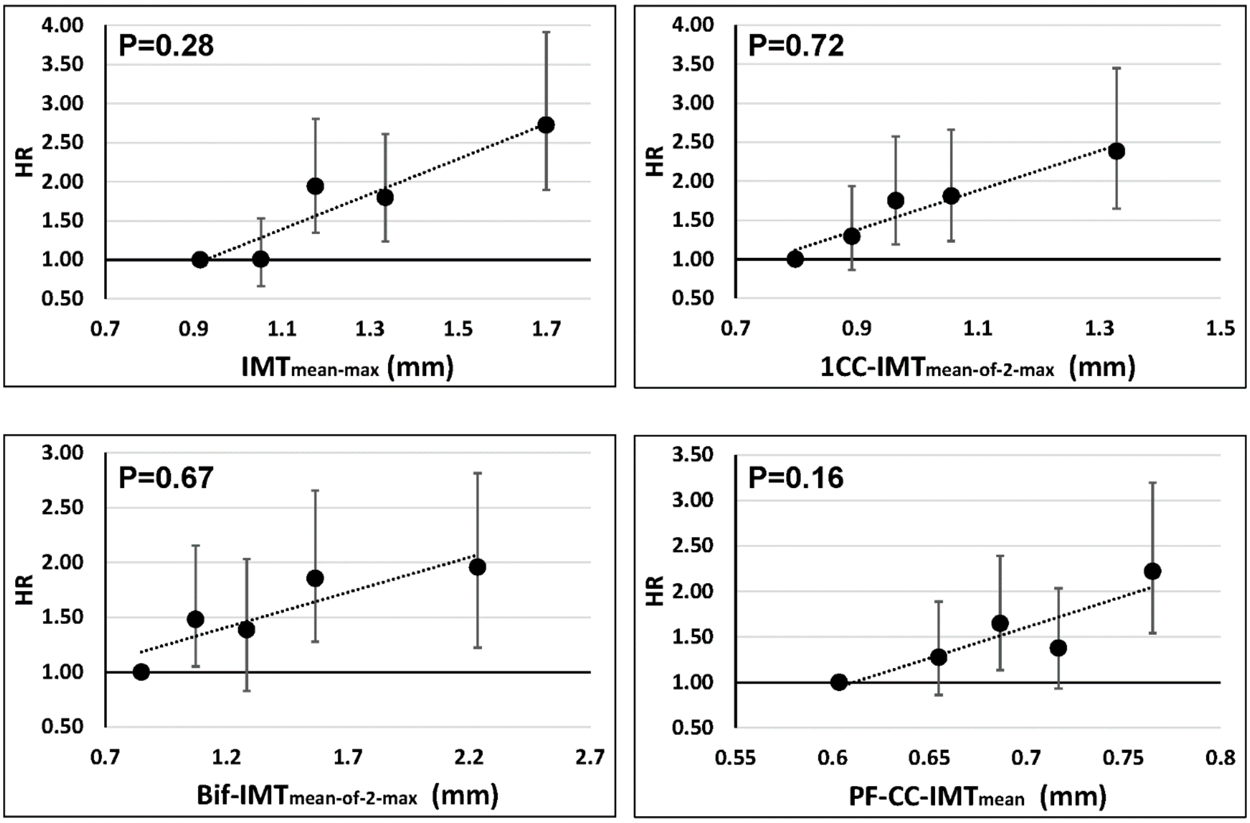

P-values for deviation from linearity tested by Martingale residuals are reported.

Quintiles boundaries of ultrasonographic variables are shown in Table S5.

Figure S3. Standardized HRs for combined events, according to different ultrasonographic variables and subgroups.

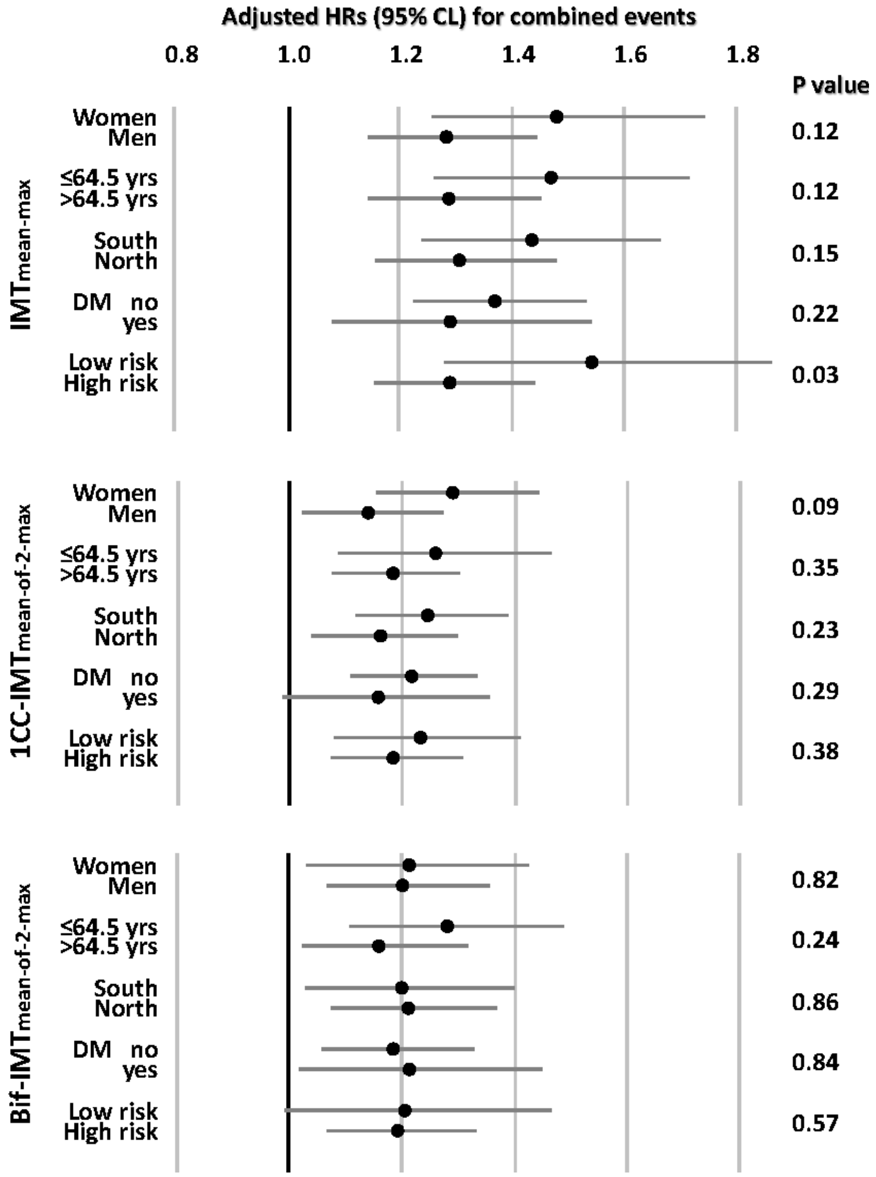

South: Italy and France; North: the Netherlands, Sweden and Finland; DM: diabetes mellitus; Low risk/High risk: baseline SCORE2/SCORE2-OP < or ≥ 8.25. Error bars are 95% Confidence Interval.

Figure S4. Calibration plots for 1CC-IMT$_{\text{mean-of-2-max}}$ and BIF-IMT$_{\text{mean-of-2-max}}$

Panel A | Panel B

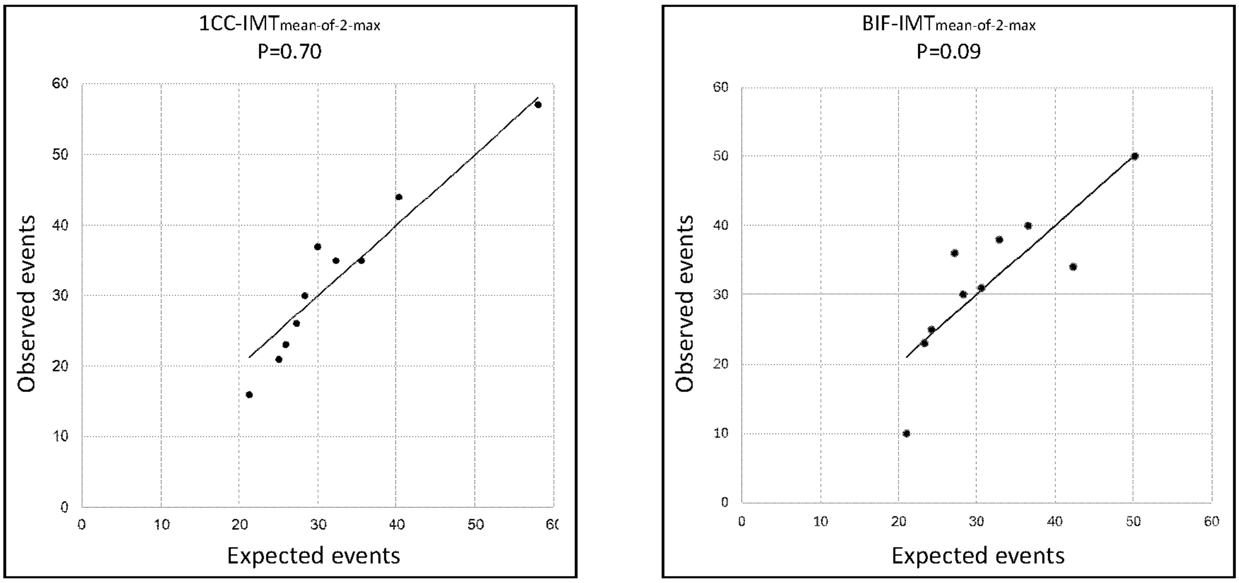

The total sample has been stratified according to deciles of 1CC-IMT$_{\text{mean-of-2-max}}$ and BIF-IMT$_{\text{mean-of-2-max}}$ and, in each stratum, the observed number of events and the number of events expected according to the logistic models were computed. The P-value refers to departure from a perfect calibration.

The IMPROVE Study Group

Centro Cardiologico Monzino, IRCCS, Milan, Italy: *Beatrice Frigerio, Daniela Sansaro, Alessio Ravani, Daniela Coggi, Alice Bonomi, Nicolò Capra, Mauro Amato, Roberta Baetta, Damiano Baldassarre.*

Department of Medical Biotechnology and Translational Medicine, Università degli Studi di Milano, Milan, Italy: *Damiano Baldassarre.*

Maria Cecilia Hospital, GVM Care & Research, Cotignola, Ravenna, Italy: *Fabrizio Veglia, Anna Malagoni, Elena Tremoli.*

Dipartimento di Scienze Farmacologiche e Biomolecolari, Università degli Studi di Milano, Milan, Italy: *Laura Calabresi, Cesare R. Sirtori.*

Department of Medicine Solna, Division of Cardiovascular Medicine, Karolinska Institutet, Stockholm, Karolinska University Hospital Solna, Sweden: *Bruna Gigante, Per Eriksson, Rona J. Strawbridge, Angela Silveira, Anders Hamsten.*

School of Health and Wellbeing, University of Glasgow, Glasgow, United Kingdom: *Rona J. Strawbridge.*

Health Data Research UK, Glasgow, UK.: *Rona J. Strawbridge.*

Division of Cardiovascular and Nutritional Epidemiology, Institute of Environmental Medicine, Karolinska Institutet: *Karin Leander, Federica Laguzzi, Ulf de Faire.*

Cardiovascular Genetics, Institute Cardiovascular Science, University College of London, Rayne Building, University Street, London, United Kingdom: *Steve E. Humphries, Jackie A. Cooper, Jayshree Acharya.*

Foundation for Research in Health Exercise and Nutrition, Kuopio Research Institute of Exercise Medicine, Kuopio, Finland: *Kai Savonen, Kirsi Huttunen, Eva Rauramaa, Ilkka M. Penttila, Jukka Törrönen.*

Department of Clinical Physiology and Nuclear Medicine, Kuopio University Hospital, Kuopio, Finland: *Kai Savonen.*

Department of Medicine, University Medical Center Groningen, Groningen & Isala Clinics Zwolle, Department of Medicine; the Netherlands: *Andries J. Smit, A.I. van Gessel, A.M van Roon, A. Nicolai, D.J. Mulder, G.H. Smeets.*

Assistance Publique - Hopitaux de Paris; Service Endocrinologie-Metabolisme, Groupe Hôspitalier Pitié-Salpetriere, Unités de Prévention Cardiovasculaire, Paris, France: *Philippe Giral, Anatole Kontush, Alain Carrié, Antonio Gallo.*

Internal Medicine, Angiology and Arteriosclerosis Diseases, Department of Medicine and Sugery, University of Perugia, Perugia, Italy: *Matteo Pirro, M.R. Mannarino, G. Vaudo, V. Bianconi, E. Marini, F. Figorilli.*

SUPPLEMENTARY REFERENCES

1. Zhuang-Zhuang, W.; Ya-Lan, F.; Xiao-Wei, W.; Chang-Xin, L.; Wei, Z.; Yang, L.; Xiao-Yuan, N. Carotid intima-media thickness in plaque-free area, carotid plaque and risk of ischemic stroke in high-risk population of North China. *Neuro Endocrinol. Lett.* **2017**, *38*, 208–214.
2. Visseren, F.L.J.; Mach, F.; Smulders, Y.M.; Carballo, D.; Koskinas, K.C.; Back, M.; Benetos, A.; Biffi, A.; Boavida, J.M.; Capodanno, D.; et al. 2021 ESC Guidelines on cardiovascular disease prevention in clinical practice. *Eur. Heart J.* **2021**, *42*, 3227–3337. https://doi.org/10.1093/eurheartj/ehab484.
